# Household presentation of influenza and acute respiratory illnesses to a primary care sentinel network: retrospective database studies (2013–2018)

**DOI:** 10.1186/s12889-020-09790-3

**Published:** 2020-11-20

**Authors:** Simon de Lusignan, Julian Sherlock, Oluwafunmi Akinyemi, Richard Pebody, Alex Elliot, Rachel Byford, Ivelina Yonova, Maria Zambon, Mark Joy

**Affiliations:** 1 Nuffield Department of Primary Care Health Sciences, University of Oxford, Woodstock Rd, Oxford, OX2 6GG UK; 2 Royal College of General Practitioners Research and Surveillance Centre, 30 Euston Square, London, NW1 2FB UK; 3 Department of Clinical & Experimental Medicine, University of Surrey, The Leggett Building, Daphne Jackson Rd, Guildford, GU2 7XP UK; 4 Public Health England, 61 Colindale Ave, London, NW9 5EQ UK

**Keywords:** Disease incidence, Infectious, Family characteristics, Population characteristics, Medical record systems, Computerized

## Abstract

**Background:**

Direct observation of the household spread of influenza and respiratory infections is limited; much of our understanding comes from mathematical models. The study aims to determine household incidence of influenza-like illness (ILI), lower (LRTI) and upper (URTI) respiratory infections within a primary care routine data and identify factors associated with the diseases’ incidence.

**Methods:**

We conducted two five-year retrospective analyses of influenza-like illness (ILI), lower (LRTI) and upper (URTI) respiratory infections using the England Royal College of General Practitioners (RCGP) Research and Surveillance Centre (RSC) primary care sentinel network database; a cross-sectional study reporting incident rate ratio (IRR) from a negative binomial model and a retrospective cohort study, using a shared gamma frailty survival model, reporting hazard ratios (HR). We reported the following household characteristics: children < 5 years old, each extra household member, gender, ethnicity (reference white), chronic disease, pregnancy, and rurality.

**Results:**

The IRR where there was a child < 5 years were 1·62 (1·38–1·89, *p* < 0·0001), 2·40 (2.04–2.83, *p* < 0·0001) and 4·46 (3.79–5.255, *p* < 0·0001) for ILI, LRTI and URTI respectively. IRR also increased with household size, rurality and presentations and by female gender, compared to male. Household incidence of URTI and LRTI changed little between years whereas influenza did and were greater in years with lower vaccine effectiveness.

The HR where there was a child < 5 years were 2·34 (95%CI 1·88–2·90, *p* < 0·0001), 2·97 (95%CI 2·76–3·2, *p* < 0·0001) and 10·32 (95%CI 10.04–10.62, *p* < 0·0001) for ILI, LRTI and URTI respectively. HR were increased with female gender, rurality, and increasing household size.

**Conclusions:**

Patterns of household incidence can be measured from routine data and may provide insights for the modelling of disease transmission and public health policy.

**Supplementary Information:**

The online version contains supplementary material available at 10.1186/s12889-020-09790-3.

## Background

Household transmission of influenza is known to be important, but its effects may be variable. Mathematical modelling, a key element of infectious disease epidemiology, makes allowance for household spread [1, 2]. and field epidemiological studies describing the household spread of influenza during 2009 pandemic [3, 4]. Serological studies indicate that a substantial proportion of household incidence may be asymptomatic, [3] younger age, particularly pre-school children [5] and female gender appears to be associated with increased household incidence; other possible correlates are household size and the presence of comorbidities [6, 7].

Some groups are known to be more susceptible to influenza and respiratory infection and therefore may be more susceptible to household transmission. These include several chronic conditions such as asthma and other chronic respiratory conditions, vascular conditions, immunosuppression [5, 8], obesity [9], and pregnancy [10].

Respiratory infections are known to be contagious and spread by droplets and contaminated fomites and therefore close proximity, such as in households, is important though evidence about the precise mechanism of transmission remain sparse [11]. Most published research focuses on specific organisms rather than on clinical conditions such as upper (URTI) and lower respiratory infections (LRTI).

We carried out this study to determine household incidence of medically attended ILI (influenza-like illness), LRTI and URTI within a routine data collected from England primary care sentinel network, and identify factors associated with the spread of the illnesses.

## Methods

### Overview

We used data from the Royal College of General Practitioners (RCGP) Research and Surveillance Centre (RSC) network, one of England’s oldest surveillance systems [12]. We carried out a 5-year retrospective analyses, firstly a repeated cross-sectional analysis comparing seasons starting 1st September 2013–14, through to 2017–18. We carried out this analysis using a negative binomial model and reporting incident rate ratio (IRR), comparing categorial variables with a defined reference (Table 3). We then carried out a retrospective cohort analyses using a frailty analysis model, reporting hazard ratios (HRs) of the influence of covariates on household incidence.

### RCGP RSC sentinel network

RCGP RSC extracts pseudonymised data from computerised medical record (CMR) systems of general practices. As the United Kingdom (UK) has a registration-based system (patients can only register with one general practitioner) this facilitates identifying incident cases. In 2013, a new database was established, and patients with the same precise address were assigned a household key. This has enabled the linkage of household members registered with the same address, and we have used this to explore the association of parental age to children with autism, [13] to look at medically attended rates of household incidence of acute gastroenteritis [14] and to report the impact of household size on coronavirus infections [15].

### Case definition

We classified a case of household incidence when two members of the same household presented on the same day ILI, LTRI or URTI or within 10 days.

We used clinical definitions of ILI, LRTI and URTI that have been used long term within the sentinel system. We define a case of ILI as an acute respiratory illness with a temperature measured/reported/plausibly ≥38 °C and cough, with onset within the past 10 days [16]. LRTI and URTI are coded as clusters of acute respiratory infections (ARI) in the RCGP. An episode of acute respiratory illness is defined as an acute pulmonary illness (including pneumonia, bronchitis and influenza-like illness) or an acute exacerbation of a chronic respiratory illness (including exacerbation of COPD, asthma or bronchiectasis).

The RCGP RSC is the national primary care surveillance systems, a long established collaboration with Public Health England (PHE), and practices are experienced at coding these conditions [17, 18]. Since 2017–2018 season, our practices receive feedback on data quality via a dashboard [19].

## Statistical methods

### Cross sectional model

Potential association between the presence of an under 5-year old in a household and transmission of influenza and respiratory illness was studied by season (2013–14 to 2017–18). Evidence for over-dispersion in transmission counts (using the Cameron-Trivedi test, [20] implemented in the R library AER, version 1.2–7 was strong (*p* < 0.001), therefore we employed a negative binomial model. We controlled for potential confounding due to gender, socio-economic status (measured by Index of Multiple Deprivation (IMD) quintile), ethnicity (reference white), presence of high risk individual in the household (see variables in Table 3), urban-rural status of the household and NHS Region and season. We maximised identification of ethnicity by using an ontology. Clinical codes were either directly mapped to ethnicity group or utilised as proxy markers (such as language spoken, and country of birth) from which ethnicity could be inferred, much routine recording of ethnicity using less-specific categories making it not possible to report below the level of white, black, Asian, mixed or other ethnicity [21]. We fitted a negative binomial model using the R library, MASS, version 7.3–45.

### Five year repeated cross-sectional study

We described the demographic features of the population including household size, the presence of a child under 5 years old in the household, comorbidities that may increase risk of spread of influenza or other respiratory infection. In addition, we utilised a typology of geographical areas in England consisting of urban, rural or town/conurbation and describe the population distribution into such areas. Based upon factors including density of population, this classification relies upon a methodology employed by a UK government agency (the Dept. for Environment, Food and Rural Affairs), published by the ONS (Office for National Statistics); see, for example, https://assets.publishing.service.gov.uk/government/uploads/system/uploads/attachment_data/file/239477/RUC11methodologypaperaug_28_Aug.pdf, [22] and differences related to urban, rural or regional location. We stratified by age, gender, deprivation (determined by converting post code at individual level to IMD quintile (the national measure of small area socioeconomic status) [23]. We divided IMD into quintiles where 1 is the most and 5 is the least deprived. Ethnicity recording was maximised using an ontological approach outlined above [21, 24]. We have also developed ontologies to maximise the detection of chronic kidney disease (CKD) [24] and pregnancy [25].

We compared the age-sex profile (ASP) for each season with the Office of National Statistics (ONS) population estimate for England 2016 (https://www.ons.gov.uk/, Supplementary data files), the ASP for household incidence, and any trend in directly standardise rate. We provide the same information for each comorbidity included in our model, and for household incidence of that condition. We separated household size with those with 2, 3, 4 or five or more occupants. We excluded single occupancy households and those with 12 or more people as they were likely to be nursing homes or old peoples’ homes.

We identified for each household covariates associated with an increased risk from influenza or lower respiratory tract infection using the categories defined by the UK Chief Medical Officer [26]. These are people with asthma and chronic respiratory disease, immunosuppression, chronic cardiovascular, liver or kidney disease, diabetes, asplenia, morbidly obese, and pregnant women. We used the World Health Organisations (WHO) classification of obesity [27].

Finally, we looked at rural-urban-conurbation differences and for regional differences between the north and south of England. To do this we categorised individuals using ONS tables, based on their post code, into those who live in rural, urban (strictly “town and city” in the ONS classification), or in conurbations. These are based on increasing population density.

### Retrospective cohort study

We studied the survival time, analysing the gap time between incidence of ILI, LRTI and URTI separately, using a shared gamma frailty model [28, 29]. We looked for incidence in households in England from 1st September 2013 until 30th April 2018. We employed a shared gamma frailty survival model with time-varying covariates to model gap times between transmission times of influenza or acute respiratory illness at the person level [28]. We used this model because over the 5 years of the longitudinal study, household transmission is a possibly recurrent event and the study population is clustered by household. The frailty term is a random effect in the model to account for the household unobserved heterogeneity. Presence of an under 5-years in the household was included in the model to study potential association with transmissions. We controlled for potential confounding at the individual level due to sex, ethnicity, age band, IMD quintile, [23] and at the household level for household size, urban-rural classification and NHS region. Age band was modelled as a time-varying covariate at the person level and household size, presence of an under 5-year old and presence of disease in a household (see: Presence of Disease in Table 4, variable types) were time-varying covariates at the household level. We used the R library FrailtySurv, v 1.3.5 [30]. We reported the results as hazard ratios, [31] together with 95% confidence intervals.

## Results

### Description of household size in the RCGP RSC network

We identified a total of 6,825,919 households. 17% of these were occupied by only one person, about 20% were occupied by 2, 3 or 4 people, 10% had 5 people living in the households while the rest (12%) had 6 or more people living in them. (Table 1).

**Table 1:** Structure of household in RCGP RSC network

Household size	Number of households	%
**1**	1,137,627	16.7
**2**	1,385,964	20.3
**3**	1,392,742	20.4
**4**	1,364,388	20.0
**5**	698,378	10.2
**6 or more**	846,820	12.4
**Total**	6,825,919	100.0

### Identifying cases of household incidence and their differing age-sex profile

We found 1407 cases of household incidence of ILI, 12,375 of LRTI and 68,503 of URTI. The number, rate, and age-sex profiles of the people affected varied from season to season in ILI, whereas the pattern people with household incidence of LRTI and URTI had similar age-sex distributions each year (Table 2). The household incidence of LRTI was bimodal with the highest rates in the under 5 and over 80-years age bands. Household incidence of URTI was also most frequent in the under 5-year old age-band with the 5- to 9-year old the next most frequent, with younger adults 25 to 45 years the group least affected. In the under 5-year olds males presented more with LRTI and URTI, whilst overall and in most age-bands females presented more than males. The same data are reported individually for each covariate included in the study (Supplementary material).

**Table 2:** Number of household presentations and rate by year of household incidence as a percentage of all ILI, LRTI and URTI cases and median age of case of household incidence

	2013–14	2014–15	2015–16	2016–17	2017–18
**ILI**					
Household presentations (n)	103	321	280	198	505
% of all cases	0.02%	0.07%	0.06%	0.04%	0.10%
Median age (IQR)	31 (36)	37 (48)	32 (35)	34 (41)	41 (43)
**LRTI**					
Household presentations (n)	2267	2769	2333	2458	2548
% of all cases	0.50%	0.58%	0.48%	0.49%	0.50%
Median age (IQR)	46 (63)	51 (58)	48 (61)	56 (53)	57 (52)
**URTI**					
Household presentations (n)	14,137	16,044	14,191	11,792	12,339
% of all cases	3.10%	3.39%	2.92%	2.37%	2.43%
Median age (IQR)	6 (29)	7 (30)	7 (28)	8 (30)	8 (29)

### Repeated cross-sectional study

The only consistent findings across all three disease areas were the associations with a child under 5-years old in the household and increasing household size. The presence of a child under 5-years gave an IRR of 1·62 (95%CI 1·38–1·89, *p* < 0·0001), 2·40 (95%CI 2·04–2·83, *p* < 0·0001) and 4·46 (95%CI 3·79–5·25, *p* < 0·001) for ILI, LRTI and URTI respectively. There was a gradation in IRR from ILI (1·62) to LRTI (2·40) to URTI (4·46). Increasing household size, had a more consistent effect on IRR. The results were for ILI 1·40 (95%CI 1·31–1·4904, *p* < 0·00010, for LRTI 1·18 (95%CI 1·11–1·26, *p* < 0·0001) and finally for URTI the IRR was 1·56 (95%CI 1·47–1·67, *p* < 0·0001, Table 3). People of Asian ethnicity had a higher IRR of presenting with household incidence of ILI and URTI, but not LRTI (compared with white ethnicity).

**Table 3:** Incident rate ratio (IRR) of household incidence - summary table for influenza-like -illness (ILI), lower respiratory infection (LRTI) and upper respiratory infection (URTI). Registered Population of the RCGP RSC Surveillance Network of primary care practices in UK. 2013/14 Season to 2017/18 Season*.* Children under 5-years in the household and increasing size were the only statistically significant incident differences across all three conditions· The IRR changed significantly between years for ILI but not for the other groups of conditions

Variable type	Variable detail	Reference group:	ILI (IRR) (95% C.I)	LRTI (IRR) (95% C.I)	URTI (IRR) (95% C.I)
**Gender**	Female: male ratio	Positive female>male	1.096 (0·98–1·22)	1·069 (0·96–1·19)	**1·159 (1·04–1·29)**
**Socioeconomic status (SES)**	IMD Quintile	Change with improved SES	0.964 (0·90–1·04)	0·993 (0·92–1·07)	**0·926 (0·86–1·00)**
**Ethnicity**	Asian	White ethnicity	**2·933 (2·20–3·90)**	1·207 (0·91–1·61)	**1·871 (1·41–2·49)**
	Black		0·549 (0·30–1·01)	0·563 (0·30–1·04)	0·796 (0·43–1·47)
	Mixed		1·368 (0·89–2·10)	0·922 (0·60–1·41)	1·020 (0·67–1·56)
	Other		1·295 (0·54–3·08)	1·081 (0·46–2·57)	1·355 (0·57–3·22)
**Preschool child**	**Under 5 in Household**	**No under 5**	**1·616 (1·38–1·89)**	**2·404 (2·04–2·83)**	**4·461 (3·79–5·25)**
**Household size**	**Household size**	**For each increase in size**	**1·399 (1·31–1·49)**	**1·182 (1·11–1·26)**	**1·563 (1·47–1·67)**
**High risk groups**	Asthma	Not present in household	0·994 (0·78–1·27)	**2·041 (1·60–2·60)**	**1·581 (1·52–1·64)**
	Immuno-supressed		1·224 (0·80–1·87)	**1·559 (1·02–2·39)**	0·781 (0·72–0·85)
	Respiratory disease		0·940 (0·58–1·53)	**2·433 (1·50–3·95)**	0·737 (0·67–0·81)
	Morbidly obese (BMI > 35)		**1·580 (1·15–2·18)**	**1·464 (1·06–2·02)**	1·360 (1·28–1·44)
	Coronary heart disease (CHD)		0·960 (0·71–1·30)	**2·051 (1·52–2·78)**	0·917 (0·87–0·97)
	Chronic kidney disease (CKD)		1·051 (0·69–1·61)	**2·196 (1·43–3·37)**	0·718 (0·65–0·79)
	Chronic liver disease		1·295 (0·98–1·72)	**1·792 (1·35–2·38)**	0·956 (0·91–1·01)
	Diabetes		1·859 (0·91–3·82)	1·399 (0·68–2·88)	1·063 (0·91–1·24)
	Asplenia		1.521 (0·78–2·95)	1·323 (0·68–2·57)	1·252 (1·11–1·42)
	Pregnancy		**0·437 (0·21–0·92)**	1·097 (0·52–2·32)	1·564 (1·48–1·66)
**ONS rural-urban conurbation**	Conurbation	City and Town	0·937 (0·65–1·35)	1·018 (0·70–1·47)	0·957 (0·66–1·38)
	Rural		**1·613 (1·25–2·08)**	1·096 (0·85–1·41)	0·886 (0·69–1·14)
**NHS Region**	Midlands and East	London	0·670 (0·44–1·03)	**1·594 (1·06–2·50)**	0·678 (0·44–1·04)
	North		**0·621 (0·43–0·90)**	**1·507 (1·04–2·18)**	0·827 (0·57–1·20)
	South		0·778 (0·51–1·18)	1·116 (0·74–1·69)	0·709 (0·47–1·08)
**Season**	2014–2015	Reference 2013–2014	**3·282 (2·20–4·89)**	1·261 (0·85–1·88)	1·115 (0·75–1·66)
	2015–2016		**2·751 (1·84–4·12)**	1·033 (0·69–1·55)	0·920 (0·61–1·38)
	2016–2017		**1·966 (1·29–3·00)**	1·055 (0·69–1·61)	0·799 (0·52–1·22)
	2017–2018		**5·385 (3·68–7·89)**	1·058 (0·72–1·55)	0·826 (0·56–1·21)

*IRR* incident rate ratio, *ILI* influenza like illness, *LRTI* lower respiratory infection, *URTI* upper respiratory infection, *C.I* confidence interval, *IMD* index of multiple deprivation, *ONS* office of the national statistics, *NHS* national health service, *sig* significance, *SES* socioeconomic status, *BMI* body mass index, *CHD* coronary heart disease, *CKD*:chronic kidney disease

Morbid obesity (WHO Class 3) was associated with increased incidence of ILI, whereas pregnancy was associated with lower rates. Asthma and the presence of an immunosuppressed person in the household was associated with a greater household incidence of LRTI and URTI. Coronary heart disease, CKD, and liver disease were associated with greater household incidence than households without these conditions. Diabetes and asplenia were not associated with a raised IRR.

Compared with London (reference region), NHS North region had a lower IRR of presentation with ILI (IRR 0·62 (95%CI 0·43–0·90, *p* = 0·011)) but was more likely to present with LRTI (IRR 1·51 (95%CI 1·04–2·18, *p* = 0·029)), though rural had a higher IRR than conurbations.

Compared with the 2013/14 (reference season), subsequent seasons had higher IRR for presentation from the same household with ILI. The differences were particularly great in the 2014/15 season (IRR 3·28 (95%CI 2·20–4·89, *p* < 0·0001) and the 2017/18 season (IRR 5·39 (95%CI 3·68–7·89, *p* < 0·0001).

Gender ratio (female over male) and IMD quintile (reduction with each quintile change towards higher socioeconomic status) were strongly associated with a higher IRR of presentation household incidence of URTI.

### Retrospective cohort study, shared frailty model

The consistent findings across the negative binomial and the survival model were that children under 5-years old and increasing household size were associated with greater household incidence of ILI, LRTI and URTI. The presence of a child under 5-years gave a HR of 2·34 (95%CI 1·88–2·90, *p* < 0·0001), 2·97 (95%CI 2·76–3·2, *p* < 0·0001) and 10·32 (95%CI (10·04–10·62), *p* < 0·0001) for ILI, LRTI and URTI respectively. There was a gradation in HR from ILI (2·34) to LRTI (2·97) to URTI (10·32). Increasing household size, also had significant effect on HR. The results were for ILI 1·56 (95%CI 1·46–1·67, *p* < 0·0001, for LRTI 1·36 (95%CI 1·36–1·41, *p* < 0·0001) and finally for URTI the HR was 1·29 (95%CI 1·27–1·30, *p* < 0·0001, Table 4).

**Table 4:** Hazard ratio (HR) of household incidence - summary table for influenza-like -illness (ILI), lower respiratory infection (LRTI) and upper respiratory infection (URTI). Registered Population of the RCGP RSC Surveillance Network of primary care practices in UK. 2013/14 Season to 2017/18 Season

Variable type	Variable detail	Reference group:	ILI (HR) (95% C.I)	LRTI (HR) (95% C.I)	URTI (HR) (95% C.I)
**Gender**	**Female:male ratio**	**female**	**0·87 (0·76–0·99)**	**0·94 (0·9–0·98)**	**0·83 (0·81–0·84)**
**Socioeconomic status (SES)**	IMD Quintile	Change with improved SES	0·91 (0·84–0·99)	0·99 (0·97–1·02)	**0·93 (0·92–0·94)**
**Ethnicity**	Asian	White ethnicity	**2·38 (2·32–2·44)**	1·06 (0·92–1·21)	1·48 (1·42–1·55)
	Black		0·81 (0·71–0·91)	**0·69 (0·53–0·9)**	**0·89 (0·83–0·95)**
	Mixed		**1·33 (1·23–1·60)**	1·04 (0·85–1·26)	**1·28 (1·20–1·37)**
	Other		1·93 (1·64–2·23)	**0·7 (0·53–0·94)**	**1·15 (1·05–1·25)**
**Preschool child**	**Under 5 in Household**	**No under 5**	**2·34 (1·88–2·90)**	**2·97 (2·76–3·2)**	**10·32 (10·04–10·62)**
**Household size**	**Household size**	**For each increase in size**	**1·56 (1·46–1·67)**	**1·36 (1·36–1·41)**	**1·29 (1·27–1·30)**
**Presence of Disease**	Asthma	Not present in household	1·05 (0·82–1·33)	**1·48 (1·4–1·58)**	**1·15 (1·11–1·19)**
	Pregnancy		**1·67 (1·03–2·70)**	0·91 (0·74–1·11)	1·03 (0·96–1·11)
	Diabetes		1·2 (0·92–1·55)	**1·31 (1·22–1·41)**	**0·7 (0·66–0·74)**
	Respiratory Condition		0·72 (0·43–1·21)	**1·43 (1·31–1·56)**	**0·53 (0·47–0·59)**
	Coronary Heart disease		1·19 (0·89–1·58)	**1·52 (1·42–1·62)**	**0·83 (0·77–0·89)**
	Chronic kidney Disease		1·14 (0·74–1·75)	**1·44 (1·32–1·55)**	**0·61 (0·53–0·69)**
	Immunosuppressed		0·99 (0·61–1·60)	**1·31 (1·14–1·50)**	**0·88 (0·78–0·99)**
	Obesity		1·48 (0·92–2·41)	**1·25 (1·09–1·32)**	**0·88 (0·80–0·97)**
	Neurological Disease		1·2 (0·82–1·75)	**1·5 (1·38–1·63)**	**0·82 (0·76–0·89)**
	Liver Disease		1·1 (0·59–2·07)	1·11 (0·87–1·41)	**0·79 (0·68–0·92)**
	Asplenia		1·36 (0·52–3·60)	0·96 (0·75–1·23)	**0·9 (0·80–1·02)**
**Urban-rural class**	Conurbation	City and Town	0·77 (0·60–0·98)	0·99 (0·99–1·07)	**0·94 (0·91–0·98)**
	**Rural**		**1·45 (1·07–1·97)**	**1·16 (1·06–1·27)**	**0·91 (0·87–0·96)**
**NHS Region**	Midlands and East	London	**0·66 (0·44–0·99)**	**1·72 (1·54–1·92)**	**0·67 (0·64–0·71)**
	North		**0·64 (0·43–0·95)**	**1·77 (1·62–1·94)**	**0·81 (0·78–0·85)**
	South		0·75 (0·51–1·10)	**1·35 (1·23–1·48)**	**0·74 (0·71–0·78)**

HR ratio, *ILI* influenza like illness, *LRTI* lower respiratory infection, *URTI* upper respiratory infection, *C.I* confidence interval, *IMD* index of multiple deprivation, *ONS* office of the national statistics, *NHS* national health service, *sig* significance

People of Asian ethnicity had a higher HR of presenting with household incidence of ILI and URTI, but not LRTI (compared with white ethnicity). HR was 2.38 (95%CI 2.32–2.44, *p* < 0.0001) for ILI and 1.48 (95%CI 1.42–1.55, *p* < 0.0001) for URTI. Rural living was significantly associated with a greater HR for household incidence of ILI and LRTI.

Asthma, diabetes, respiratory disease, coronary heart disease, CKD, obesity, Neurological disease and the presence of an immunosuppressed condition were associated with a significant household incidence of LRTI and URTI. Pregnancy was associated with a raised HR of presentation of household transmission of ILI, this was one of the few areas of contradiction between our results.

Female gender and lower SES were associated with a greater HR of household incidence of URTI in both models, in our frailty model female gender was associated with higher rates of household presentation with all three conditions.

## Discussion

### Principal findings

**Fig. 1:**
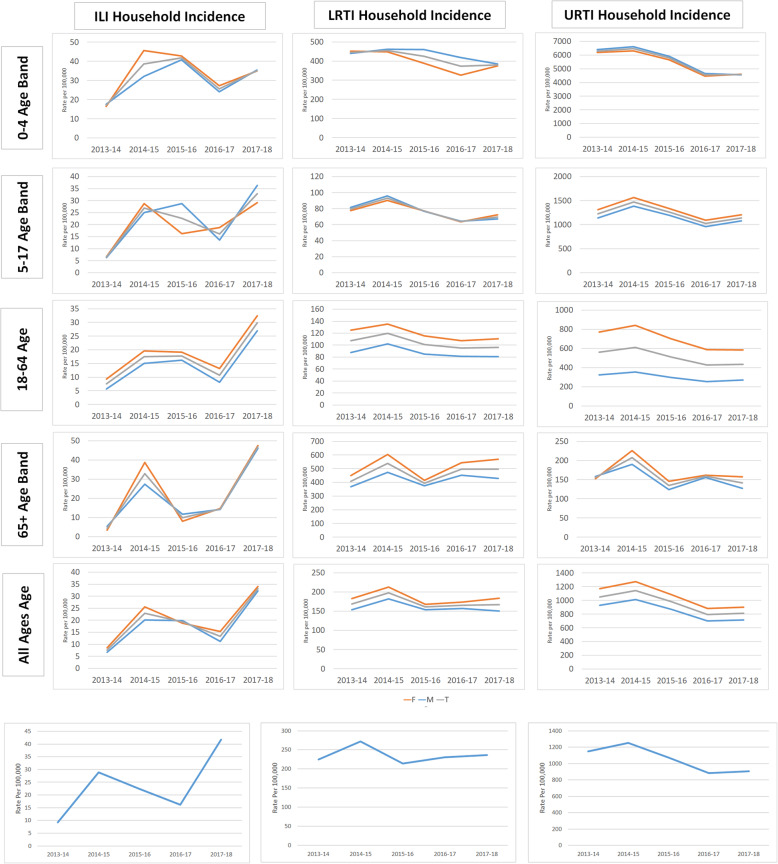
Standardised rates of household incidence cases for ILI, LRTI, URTI by age band, gender and year. There is most variation between years in ILI, though LRTI and URTI follow a similar pattern. Other than for some years in ILI and in LRTI and URTI in the 0-4 year age-band, females generally present more than males. Change in incidence rate of ILI, LRTI and URTI with household size, socioeconomic status and presence of children under 5-years in the household: Registered Population of the RCGP RSC Surveillance Network of primary care practices in UK. 2013/14 Season to 2017/18 Season

**Fig. 2:**
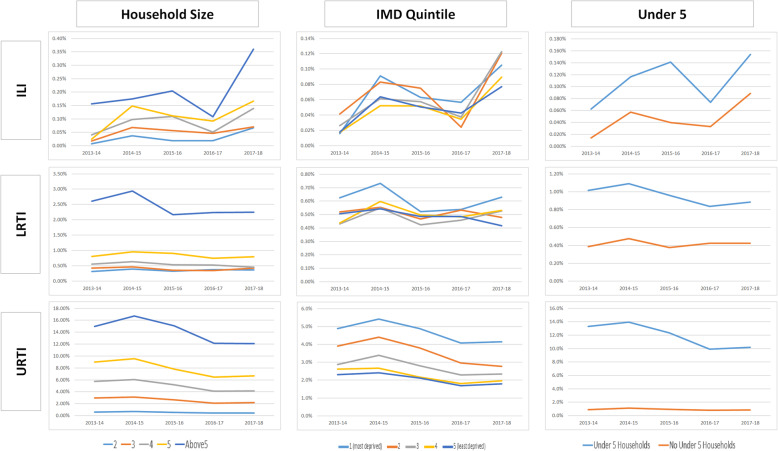
Association of household size, socioeconomic status and a child under 5-years old in the household on household incidence rates of ILI, LRTI and URTI. Registered Population of the RCGP RSC Surveillance Network of primary care practices in UK. 2013/14 Season to 2017/18 Season

We identified household incidence from direct analysis of routine primary care data. The highest rate was seen in URTI, then lower rates for LRTI and then ILI, respectively. The age-bands presenting in LRTI and URTI did not change greatly year-on-year, however they vary in ILI (Figs. 1 and 2, and Table 2). In years when ILI had higher levels of household incidence there were also increased rates of presentation of LRTI and URTI.

### Change in standardised rates

The standardised rates of household incidence had a similar pattern each year by age-band. However, there was variation between years with the difference much greater between years in ILI. Generally, females overall and across all ages presented more ILI, LRTI and URTI – other than in the under 5-years old category where boys presented more with household incidence of LRTI and URTI; and similarly, in the age 5 to 17-years age band, males presented more with household incidence of ILI and LRTI (Fig. 1).

A child under 5- years old in the household and increasing household size were associated with increased household incidence of all three conditions. The ranking of increased incidence from ILI, to LRTI to URTI was consistent between our models.

In our observational data, males presented more than females with ILI in some years, though boys under 5 years old consistently more with LRTI, and URTI (Fig. 1). Overall, as in our frailty model, females had a higher incidence than males (Table 4). Households with a person with comorbidities associated with increasing risk from influenza had a higher IRR and HR of household incidence of LRTI but not ILI (Tables 3 and 4) but a lower HR of URTI was seen in this group (Table 4). Asthma, though, was an exception to this.

Rural residence was associated with greater household incidence of ILI (both models) and LRTI (frailty), and again a lower incidence of URTI (frailty model, Tables 3 and 4). A contrasting pattern was seen in household incidence of ILI between NHS regions with London, regions outside London had lower rates of ILI. However, regions outside London generally had a higher rate of household incidence of LRTI, though lower rates of URTI.

### Implications of the findings

Infections presenting within the same household can be identified in routinely collected CMR system data. The rate of household incidence of ILI varied and was highest in years where the rates were elevated in the general population, as in 2014/15 and 2017/18; seasons when there was either an antigenic or B-lineage mismatch to that season’s influenza vaccine. Household incidence data could be used to supplement other measures of vaccine effectiveness [32] (Fig. 2); including before and after the introduction of respiratory syncytial virus (RSV) vaccine.

Households with a child under 5-years of age and increasing household size were unsurprisingly, associated with higher rates of household incidence. This reinforces the view that young children are the carriers of many respiratory illnesses and supports the rationale for flu and other respiratory vaccinations in childhood [33]. Likewise increasing household size is an anticipated finding, as public health messages or invitations for vaccination were linked to household size [26] The differences between genders are interesting. The standardised crude data from our cross-sectional study (Fig. 1) reinforced findings in a previous RCGP RSC annual report paper that boys present more than girls with acute respiratory infections [17].

It was interesting that there was no greater presentation of household incidence of ILI from households with high risk people, but there was of LRTI but a lower HR of URTI. In a previous study although we reported more ILI and LRTI in diabetes, whilst presentation did not increase with poor diabetes control [34]. The lowest HR for URTI was from households with existing respiratory disease. However, asthma did not fit this pattern, it was the only comorbidity with a higher HR of presenting with household incidence of URTIs. This is possibly because asthma is a familial condition and can be precipitated by viral URTI.

We did not find any clear message from comparing regions or rurality. It would appear that rural and London regions have more household presentation with ILI, as seen in both models. However, in our frailty model, we showed more LRTI and less URTI in regions outside London.

### Comparison with the literature

Our findings fit with existing evidence relating to pre-school children, female gender, and household size [6]. Whilst it is possible that household incidence in larger households might be due to increased contacts, another contributor is that children under 5-years shed flu virus for longer [35].

The presence of comorbidities in our study was not associated with increased household presentation of ILI or URTI (other than for households with asthma), though it was for LRTI, this contrasts with findings from other studies [6, 9]. Our frailty model suggested a higher HR of household transmission of ILI in pregnancy and supported by the literature, [10] though this was not found in our cross-sectional analysis. It is possible this difference was due to factors included in our frailty model but not in the cross sectional analysis. Additionally, immunisation programmes across the course of our study may have had an impact and should be considered in future studies. Pregnant women in the UK are immunised against influenza following the pandemic of 2009, to protect them from the increased risk of severe infection [36] and infants in the UK were also offered the pneumococcal conjugate vaccine (PCV7) in September 2006, followed by the PCV13 in April 2010 [37].

### Strengths and limitations

This study was conducted using data from a well-established sentinel network, ending its 52nd season. Practices contribute data to a weekly report and receive feedback about data quality. Whilst our data have limitations, we feel RCGP RSC data are as good as they get from routine primary care CMR systems. Household incidence may represent household transmission, we decided that we would use the term “household incidence” as it is possible that cases within the same household were a result of school or other shared exposure. We have not looked at the effect of vaccines and more complex modelling on household incidence of respiratory illnesses; these will be considered as part of future research. The findings reported in this paper are based on codes from CMR systems, we have no virological or other laboratory / independent confirmation of diagnoses; virological sampling in RCGP RSC is restricted to 100 practices and only through the influenza season. Another limitation of our study is possible underestimation due to health care seeking as asymptomatic people may not visit their GPs. We also note that our ethnic categories are limited to those available widely in our source data, and that more granular ethnic data would be desirable in any future study.

### Call for further research

Further research including vaccine records and virological specimens would provide a greater understanding of the nature of household incidence. It is possible there may be indirect benefits of influenza vaccination in households [36, 37]. Virological studies could also elicit the nature of the organism and whether our household incidence cases are genuinely household transmission.

We are able to identify communal establishments in our data, and identify those including older people. A study of this type would be useful, but beyond the scope of this paper.

Lastly, this methodology may be adapted to better understand the transmission of COVID-19 within households; the findings of such a study would inform policies regarding the reopening of schools and workplaces.

## Conclusion

Household incidence can be detected from routine data collected in a sentinel network. This study reports from analysis of routine clinical data how household size and children under 5-years old are associated with a higher incidence of household presentation of influenza and other respiratory diseases. Our study shows that the risk of ILI, LRTI or URTI for people living in households with children under 5 years can be twice, thrice or ten times higher respectively than people living in households without children under 5 years. In addition, people living in larger households have a higher risk of infection with respiratory illnesses. These results align with previous research in household transmission studies. Younger age and number of household contacts have appeared in many recent studies, which report their association with higher susceptibility, see Table 1 in [37].

Although not shown in our study, greater incidence might provide a signal of reduced vaccine effectiveness in a particular season. It highlights that vaccination of young children against influenza may be pertinent in reducing transmission and further investigation including exposure to vaccines are needed. Ongoing direct measurement of household incidence may provide further insights into the epidemiology of respiratory infections and household composition and size. This might be a useful component of programmes for targeting vaccine update.

## Supplementary Information

**Additional file 1.**

## Data Availability

All data analysed or generated during the study are included in the submission. The datasets however, are not available publicly, but can be made available upon request from the corresponding author.

## Abbreviations

CKD: Chronic Kidney Disease
CMR: Computerized medical record
GP: General Practice
HR: Hazard ratio
ILI: Influenza-like illness
IMD: Index of multiple deprivation
IRR: Incident rate ratio
LRTI: Lower respiratory tract infections
NHS: National Health Service
PHE: Public Health England
PCV: Pneumococcal conjugate vaccine
RCGP: Royal College of General Practitioners
RSC: Research and Surveillance Centre
SES: Socioeconomic status
UK: United Kingdom
URTI: Upper respiratory tract infections
WHO: World Health Organisation

## References

[1] Riley S (2007). Large-scale spatial-transmission models of infectious disease. Science..

[2] Grassly NC, Fraser C (2008). Mathematical models of infectious disease transmission. Nat Rev Microbiol.

[3] Lau LL, Nishiura H, Kelly H, Ip DK, Leung GM, Cowling BJ (2012). Household transmission of 2009 pandemic influenza a (H1N1): a systematic review and meta-analysis. Epidemiology..

[4] Cauchemez S, Donnelly CA, Reed C, Ghani AC, Fraser C, Kent CK, Finelli L, Ferguson NM (2009). Household transmission of 2009 pandemic influenza a (H1N1) virus in the United States. N Engl J Med.

[5] Viboud C, Boëlle PY, Cauchemez S, Lavenu A, Valleron AJ, Flahault A, Carrat F (2004). Risk factors of influenza transmission in households. Br J Gen Pract.

[6] Tsang TK, Lau LL, Cauchemez S, Cowling BJ (2016). Household transmission of influenza virus. Trends Microbiol.

[7] McCaw JM, Howard PF, Richmond PC (2012). Household transmission of respiratory viruses–assessment of viral, individual and household characteristics in a population study of healthy Australian adults. BMC Infect Dis.

[8] Hall C (2007). The spread of influenza and other respiratory viruses: complexities and conjectures. Clin Infect Dis.

[9] Gilca R, De Serres G, Boulianne N, Ouhoummane N, Papenburg J, Douville-Fradet M, Fortin E, Dionne M, Boivin G, Skowronski DM (2011). Risk factors for hospitalization and severe outcomes of 2009 pandemic H1N1 influenza in Quebec, Canada. Influenza Other Respir Viruses.

[10] Mertz D, Geraci J, Winkup J, Gessner BD, Ortiz JR, Loeb M (2017). Pregnancy as a risk factor for severe outcomes from influenza virus infection: a systematic review and meta-analysis of observational studies. Vaccine..

[11] Kutter JS, Spronken MI, Fraaij PL, Fouchier RA, Herfst S (2018). Transmission routes of respiratory viruses among humans. Curr Opin Virol.

[12] Correa A, Hinton W, McGovern A, van Vlymen J, Yonova I, Jones S, de Lusignan S (2016). Royal College of general practitioners research and surveillance Centre (RCGP RSC) sentinel network: a cohort profile. BMJ Open.

[13] Hoang U, James AC, Liyanage H, Jones S, Joy M, Blair M, Rigby M, Lusignan S (2019). Determinants of inter-practice variation in ADHD diagnosis and stimulant prescribing: cross-sectional database study of a national surveillance network. BMJ Evid Based Med.

[14] de Lusignan S, Konstantara E, Joy M (2018). Incidence of household transmission of acute gastroenteritis (AGE) in a primary care sentinel network (1992-2017): cross-sectional and retrospective cohort study protocol. BMJ Open.

[15] de Lusignan S, Dorward J, Correa A, Jones N, Akinyemi O, Amirthalingam G, Andrews N, Byford R, Dabrera G, Elliot A, Ellis J, Ferreira F, Lopez Bernal J, Okusi C, Ramsay M, Sherlock J, Smith G, Williams J, Howsam G, Zambon M, Joy M, FDR H. Risk factors for SARS-CoV-2 among patients in the Oxford Royal College of General Practitioners Research and Surveillance Centre primary care network: a cross-sectional study. Lancet Infect Dis. 2020. 10.1016/S1473-3099(20)30371-6. 10.1016/S1473-3099(20)30371-6 PMC7228715 32422204

[16] Fitzner J, Qasmieh S, Mounts AW, Alexander B, Besselaar T, Briand S, Brown C, Clark S, Dueger E, Gross D, Hauge S, Hirve S, Jorgensen P, Katz MA, Mafi A, Malik M, McCarron M, Meerhoff T, Mori Y, Mott J, Olivera MTDC, Ortiz JR, Palekar R, Rebelo-de-Andrade H, Soetens L, Yahaya AA, Zhang W, Vandemaele K (2018). Revision of clinical case definitions: influenza-like illness and severe acute respiratory infection. Bull World Health Organ.

[17] de Lusignan S, Correa A, Pebody R, Yonova I, Smith G, Byford R, Pathirannehelage SR, McGee C, Elliot AJ, Hriskova M, Ferreira FI, Rafi I, Jones S (2018). Incidence of Lower Respiratory Tract Infections and Atopic Conditions in Boys and Young Male Adults: Royal College of General Practitioners Research and Surveillance Centre Annual Report 2015–2016. JMIR Public Health Surveill.

[18] Pebody RG, Whitaker H, Ellis J, Andrews N, Marques DFP, Cottrell S, Reynolds AJ, Gunson R, Thompson C, Galiano M, Lackenby A, Robertson C, O'Doherty MG, Owens K, Yonova I, Shepherd SJ, Moore C, Johnston J, Donati M, McMenamin J, Lusignan S, Zambon M. End of season influenza vaccine effectiveness in primary care in adults and children in the United Kingdom in 2018/19. Vaccine. 2020;38(3):489–497. doi: 10.1016/j.vaccine.2019.10.071. Epub 2019 Nov 1. PubMedPMID: 31685296.

[19] Pathirannehelage S, Kumarapeli P, Byford R (2018). Uptake of a dashboard designed to give Realtime feedback to a sentinel network about key data required for influenza vaccine effectiveness studies. Stud Health Technol Inform.

[20] Cameron AC, Trivedi PK (1990). Regression-based tests for overdispersion in the Poisson model. J Econ.

[21] Tippu Z, Correa A, Liyanage H (2017). Ethnicity Recording in Primary Care Computerised Medical Record Systems: An Ontological Approach. J Innov Health Inform.

[22] Bibby P, Henneberry J, Halleux JM (2020). Under the radar?‘Soft’residential densification in England, 2001–2011. Environ Plan B Urban Analytics City Sci.

[23] English_Indices_of_Deprivation_2015_-_Frequently_Asked_Questions_Dec_2016.pdf URL: https://assets.publishing.service.gov.uk/government/uploads/system/uploads/attachment_data/file/579151/. Accessed 20/08/18.

[24] Cole NI, Liyanage H, Suckling RJ (2018). An ontological approach to identifying cases of chronic kidney disease from routine primary care data: a cross-sectional study. BMC Nephrol.

[25] Liyanage H, Williams J, Byford R, de Lusignan S. Ontology to identify pregnant women in electronic health records: primary care sentinel network database study. BMJ Health Care Inform. 2019;26(1). pii: e100013. doi:10.1136/bmjhci-2019-100013. 10.1136/bmjhci-2019-100013 PMC7062332 31272998

[26] Chief Medical Officer. The national flu immunisation programme letter. 2019/20. London, Department of Health and Social Care, Public Health England, NHS England. URL: https://assets.publishing.service.gov.uk/government/uploads/system/uploads/attachment_data/file/694779/Annual_national_flu_programme_2018-2019.pdf. Accessed 1/10/19.

[27] World Health Organisation (WHO). Obesity: preventing and managing the global epidemic. Report of a WHO Consultation (WHO Technical Report Series 894) www.who.int/nutrition/publications/obesity/WHO_TRS_894/en/ accessed March 2020. 11234459

[28] Rondeau V, Filleul L, Joly P (2006). Nested frailty models using maximum penalized likelihood estimation. Stat Med.

[29] Rondeau V, Mathoulin-Pelissier S, Jacqmin-Gadda H, Brouste V, Soubeyran P (2007). Joint frailty models for recurring events and death using maximum penalized likelihood estimation: application on cancer events. Biostatistics..

[30] Rondeau V, Mazroui Y, Gonzale J (2012). Frailtypack: An R Package for the Analysis of Correlated Survival Data with Frailty Models Using Penalized Likelihood Estimation or Parametrical Estimation J. Stat Softw.

[31] Sedgwick P, Joekes K (2015). Interpreting hazard ratios. BMJ.

[32] Pebody RG, Green HK, Warburton F (2018). Significant spike in excess mortality in England in winter 2014/15 - influenza the likely culprit. Epidemiol Infect.

[33] Rondy M, Kissling E, Emborg HD, et al. I-MOVE/I-MOVE+ group. Interim 2017/18 influenza seasonal vaccine effectiveness: combined results from five European studies. Euro Surveill. 2018;23(9). 10.2807/1560-7917.ES.2018.23.9.18-00086. 10.2807/1560-7917.ES.2018.23.9.18-00086 PMC5840921 29510782

[34] Hine JL, de Lusignan S, Burleigh D (2017). Association between glycaemic control and common infections in people with type 2 diabetes: a cohort study. Diabet Med.

[35] Ng S, Lopez R, Kuan G (2016). The timeline of influenza virus shedding in children and adults in a household transmission study of influenza in Managua, Nicaragua. Pediatr Infect Dis J.

[36] Dabrera G, Zhao H, Andrews N, Begum F, Green H, Ellis J, Elias K, Donati M, Zambon M, Pebody R (2014). Effectiveness of seasonal influenza vaccination during pregnancy in preventing influenza infection in infants, England, 2013/14. Euro Surveill.

[37] Rodrigo C, Bewick T, Sheppard C, Greenwood S, Mckeever TM, Trotter CL, Slack M, George R, Lim WS (2015). Impact of infant 13-valent pneumococcal conjugate vaccine on serotypes in adult pneumonia. Eur Respir J.

